# Proposal for the Integration of the Assessment and Management of Electrical Risk from Overhead Power Lines in BIM for Road Projects

**DOI:** 10.3390/ijerph192013064

**Published:** 2022-10-11

**Authors:** Darío Collado-Mariscal, Juan Pedro Cortés-Pérez, Alfonso Cortés-Pérez, Antonia Cuevas-Murillo

**Affiliations:** 1 Department of Construction, School of Technology, University of Extremadura, Avda. de la Universidad s/n, 10003 Cáceres, Spain; 2 Department of Education, Faculty of Legal and Economic Sciences, University Isabel I, C. de Fernán González nº 76, 09003 Burgos, Spain; 3 AC2 Innovación, S.L., C/Santa Cristina, 3, Despacho B07, 10195 Cáceres, Spain

**Keywords:** 3D visualization, BIM, overhead power lines, risk assessment, risk management methodology, 4D risk simulation

## Abstract

Electrical risk has a particular impact within the construction sector. This leads to the development of regulations to mitigate it, but correct application of regulations is impossible with a traditional 2D analysis. The construction sector is using technologies from the industrial sector (Construction 4.0), with BIM as the main enabling technology. Thus, the objective of this article is the evaluation of the risk produced by Overhead Power Lines (OPL) through BIM integration. The OPL, its risk zones, the affected road, and the envelope resulting from the geometry of the necessary machines to build it were digitized, converging in a single model to perform a 4D risk analysis. The risks of the execution of the embankment and road surface of a road section passing through an OPL were analyzed by means of the collision of the envelope with its risk zones, resulting in an integration of their evaluation, to which was added the introduction of preventive measures and their re-evaluation. The parametric 3D modelling allowed a better definition of the risk zones and the BIM management minimized errors, providing traceability of decisions from the design phase, complying with health and safety regulations and applying the principle of Prevention through Design (PtD).

## 1. Introduction

The construction industry is one of the most dangerous sectors of the economy. For example, according to the U.S. Bureau of Labor Statistics, the highest number of occupational fatalities in 2020 occurred in the construction industry [1].

Although there have been significant improvements in construction technologies, the occupational accident rate in construction remains one of the highest [2]. For this reason, the prevention of workers’ health and safety hazards and risks has been and is analyzed by numerous organizations, such as [3] in the European Union or [1] in the United States, which has given rise to a broad regulatory framework consolidated as the Occupational Safety and Health Administration (OSHA), or at the European level [4] from which the laws of the countries emanate, such as [5].

Given the linear development of a road, services such as the Overhead Power Line (OPL) are affected during its construction [6].

This number increases closer to populated areas. The interaction with OPL gives rise to electrical risk, one of the risks with the most serious consequences in the construction sector, with electrocution being one of the causes of fatal accidents [7]. Furthermore, according to Asady et al. [8] the average number of potential years of life lost due to electric shocks in occupational accidents is 40 years. In the same sense, Im et al. [9] found that 9.6% of deaths studied in the construction sector are due to electric shock, while in other sectors they are 4.8%. Taylor carried out an analysis of electrocutions and found that the greatest cause, 41.6%, was due to impact with OPL [10].

Awareness of the problem has led to standards at the international level, for example, in the U.S. the IEEE 1584 and NFPA 70E [11]. These standards identify safe work practices to protect workers from the hazards of electricity: electric shock, electrocution, arc flash, and arc blast [12]. At the European level, directives have been developed such as [13], which are materialized at the national level in laws and guides such as, for example, in Spain [14], or technical guides such as those of the National Institute for Safety and Health at Work, [15] or [16].

On the other hand, industry is immersed in its digitalization under the framework of Industry 4.0, which in construction gives rise to Construction 4.0 [17]. This environment implies complexity, which in the case of health and safety requires new paradigms and assessment methods [18]. In this transformation, Building Information Modelling (BIM) technology is the enabling technology for Construction 4.0 [19]. This is reflected in the research conducted by Forcael et al. [20], which indicates that the integration of BIM in Construction 4.0 means a more efficient flow of information during the construction process. The work by Conrad et al. [21] demonstrates that BIM plays a key role in the digital transformation of construction.

The use of BIM in design helps to manage the complexity of digital construction processes [22]. Although the term Civil Information Modelling (CIM) is sometimes used [23] for the case of infrastructure, the term BIM is more widely used and will be used in this research. An example of the great progress being made in the application of BIM is the development of regulations such as the European directives [24] or in the case of road design, the actions of different countries such as Great Britain [25] or Germany [26].

Authors such as Aziz and Kumar [27,28] highlight the benefits of BIM adoption in infrastructure construction, including health and safety.

There is a consolidated international regulatory framework for the integration of safety and health in non-BIM road projects [29], with electrical risk assessment methodologies. However, this is not the case for projects developed with BIM, where the only standard that exists is that of the British Standards Institution [30], which is generalist, and does not detail how to carry out electrical risk assessment in road projects.

This situation means not being able to apply the prevention through design (PtD) methodology to avoid or mitigate risk in the design phase [31] and not being able to obtain the benefits of BIM for prevention management [32].

Thus, the aim of the study is a proposal for the integration of the assessment and management of electrical risk due to OPL in road projects carried out with BIM that makes it possible to obtain the benefits of this technology for the health and safety of workers, avoiding the emergence of new risks due to the lack of coordination between a road design in BIM and the health and safety that is not in BIM.

## 2. State of the Art

### 2.1. Risk Characterisation of Electrical Risk in Road Construction

Electrical hazards are caused not only by contact with the element, but also by generation of the electric arc. The American National Fire Protection Association (NFPA) describes a dangerous condition caused by the electric arc [33], so the risk exists at a certain distance from the OPL, not being visible, which makes it difficult to detect, as well as to raise awareness among workers. The INSST guide NTP 72 [34] analyses the prototype accident due to an element at height: dump truck, pump truck, mobile cranes, etc., due to contact or excessive proximity to OPL, determining that, in addition to death, the damage caused is very serious, such as amputations of limbs or burns.

Malekitabar et al. [35] report that electrocuted workers in the construction sector outnumber those in all other industries. The incidents are due to long metal objects such as hoists and cranes on construction projects, coupled with a poor understanding of the actual distances between these objects and the OPL.

Regulations such as IEEE 1584 and guides such as [15] are based on tables and/or 2D diagrams to delimit risk zones according to the distance to the power line and its voltage, among other factors [34]. This guide contains 2D diagrams that relate machinery to the OPL. The geometric development of road works and the curved layout of the OPL make the correct application of these guides and therefore correct risk management impossible. This makes it even more difficult to set out preventive measures on site.

The complexity of the road layout and the layout of the OPL results in this interference being one of the factors of delays in the execution of roads [36,37], with consequent health and safety risks and cost overruns, [38] or [39]. These delays are caused by the lack of location of services or lack of definition in the plans [40], which is often due to the lack of vertical information [41]. Similar conclusions can be drawn from the study carried out by Iberdrola Electric Company [42] in which more than 94 % of failures in electrical networks in Spain have their origin in the failure to detect the presence of electrical networks during execution, the failure to assess the risk, and the failure to plan safety measures.

### 2.2. Digitization in the Road Project

The information structure of linear infrastructure in BIM models differs substantially from that of buildings, which requires specific processes [43]. An example of this is that the information exchange standard (IFC) is almost two decades old, but road information was not incorporated until less than three years ago, as anticipated [44].

Despite the above, the use of BIM in road design is advancing in recent years [45]. In China, there has been interest in municipal road design BIM by combining it with 2D CAD drawings to share digital design information [46]. Lou et al. [47] studied the application of BIM to complex urban environments, highlighting its value in ensuring project safety and quality.

BIM has contributed to the real time evaluation of design criteria and rule checking and has contributed to design quality [48]. This author also detects that BIM can help in the different design phases, and in the simulation of the planning of the work in a visual way (4D).

In the design phase of a road, BIM assists in the evaluation of design criteria and design quality, planning, cost estimation, as well as finding and resolving interferences with affected services [48]. In the earthworks phase, Raza et al. [49] analyze the connection of the 3D model with the site planning (4D) interference construction phase with OPL. This 3D analysis capability is investigated by Bae et al. [50] to optimize road pavement repairs with centimeter accuracy.

Light Detection and Ranging (LiDAR) technology is increasingly used for the digitization of the environment in road projects [48], for the creation of the Digital Surface Model [51], and for the monitoring of excavations [52]. This digitization of the environment is being integrated into BIM for better information management [53].

LiDAR is also being applied to obtain the geometry of existing OPL. Thus, Guo et al. [54] have developed a method for the detection of OPL cables and poles using LiDAR data. This allows for accurate georeferenced models of the entire OPL. The same objective was pursued by the research of Zhao et al. [55] by developing an algorithm to detect OPL from photographs taken with unmanned aerial vehicles. These 3D models of power lines are used for, among others, risk detection based on the definition of the catenary curve describing the cable [56].

BIM technology, being based on parametric design, allows automation, applying object-based programming tools such as Dynamo 2.3. Tang et al. [57] used it to automate 3D road pavement model generation and analysis. Sheikhkhoshkar et al. [58] also used it to automate 4D simulation from Revit 2020 models.

### 2.3. Health and Safety and BIM

Costin [48], indicates that the integration of BIM in infrastructure projects is a tool for the systematic management of occupational risks to control them and improve health and safety. Moreover, the use of BIM would allow the planning of activities to avoid space conflicts. Martínez-Aires et al. [59] highlight the advantage of BIM in visualization for detecting and controlling occupational risks. In addition to this, there are advantages for prevention, as realistic simulations can be carried out [60].

The integration of Health and Safety in BIM has been addressed by different authors with different approaches. Thus, Ganah, Jin, and Musa [60,61,62] establish a general conceptual framework based on visualization to improve health and safety practices on site, without advancing specific aspects of infrastructures. In other cases, a partial proposal is made, such as Zhang et al. [63] when addressing the risk of falling through gaps in buildings. On the other hand, Getuli et al. [64] proposes the implementation through the creation of design verification and validation rules within a BIM environment, through the parameterization of Health and Safety regulations as well as the creation of BIM objects with safety requirements. Other authors such as Ganah [60] focuses the use of BIM on the communication of health and safety and the visualization of risks through a color code depending on the severity of the risk as did Shen et al., for construction risks [65], Fernández-Rodríguez et al. [66] for allergy risk in buildings, or Fernández-Alvarado et al. [67] representing the risk of pollen on the roadway and pavement of a road.

The research by Hossain et al. [68] represents a significant advance by proposing an intelligent risk identification and management system integrated in BIM based on a library with logical rules to help designers identify risks. This proposal is designed for the construction of buildings and does not take into account the particularities of roads and power lines.

Malekitabar et al., propose a significant improvement for risk management in BIM using risk controllers [35]. In the case of electrical risks, he identifies eight control rules, some of which are: existence, identifying its position in the model; proximity, indicating the distance to the power lines; establishing the route to be taken by a scaffold; or pathfinding, with possible states of a crane and its boom.

In addition to the 3D model, different authors have proposed the importance for health and safety of developing 4D models including the planning of construction tasks, Zhang et al. [63].

In the study proposed by Vahdatikhaki [69], the risks produced in the radius of action of machinery in the development of earthmoving works are analyzed in order to study the interdependencies between activities, establishing a direct relationship with the site plan and with the BIM model.

Lee et al. [70], in addition to identifying risks, evaluates them through their probability and severity, obtaining risk output parameters, but applied to buildings and without considering the electrical risk. Cortés-Pérez et al. [32] propose a methodology that encompasses the entire process from risk assessment to reassessment in a single model, which guarantees the traceability of the process, but in building projects carried out in BIM.

The only work that addresses occupational risk assessment in roads using BIM is by Zou et al. [71]. This work focuses on the establishment of the communication system for safety and health management in road projects, and for the construction phase. It does not cover the design phase and the process from risk assessment to risk re-evaluation within the BIM model.

## 3. Materials and Methods

### 3.1. Initial Information and Road Modelling

**Figure 1:**
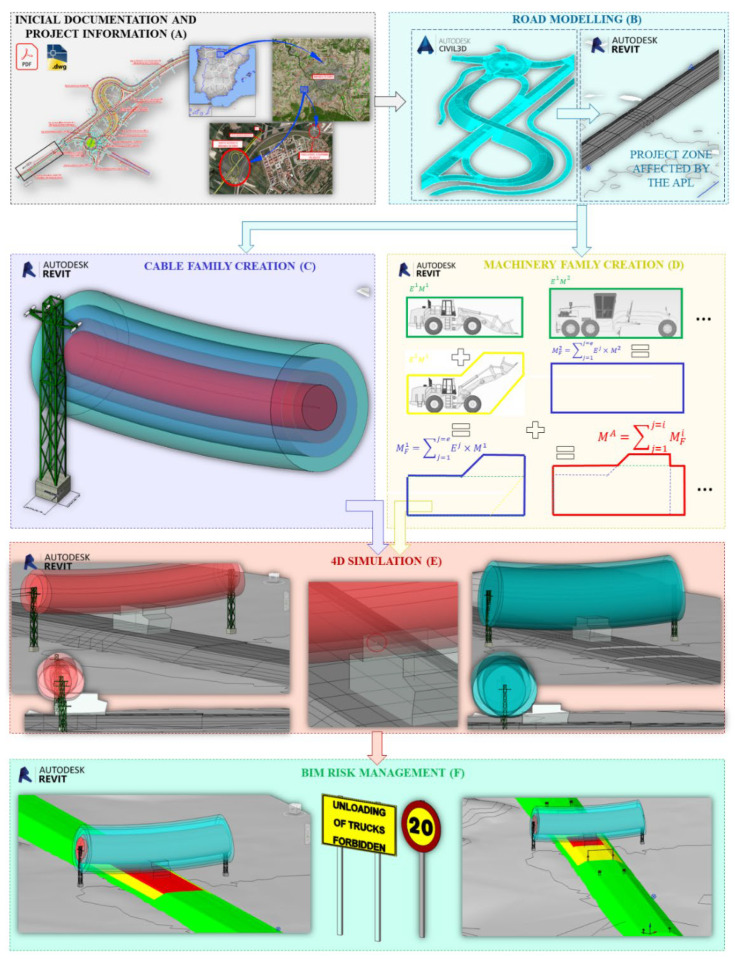
General outline of the methodology.

Based on the complete road project (Figure 1A), the area around the OPL has been measured for this investigation using the Civil 3D 2020 tool, at a distance which is consistent with the regulations for construction site markings [72]. The study area is discretized horizontally in sections by zones, and vertically according to the different layers of material and the phases of execution of the construction process. The discretized road model is exported to Revit with shared coordinates, with the model for the health and safety analysis having the same coordinates as the Civil 3D model. The topography is also introduced into the model by means of the Revit 2020 Site Designer Plugin in shared coordinates, completing the representation of the work in the OPL environment (Figure 1B).

### 3.2. Geometric Modelling of the OPL

Based on the geometry of the OPL layout, whose position and geometry are known and is a reference to the topography of the project, the georeferenced BIM objects that simulate the pylons in the position established in the original project are introduced into the Revit model. From these data the OPL is created as a catenary curve, for which the coordinates of the start and end points, which are given by the towers, are necessary. To determine the cable equilibrium configuration, the cable equilibrium configuration equations are used in the hypothesis of a homogeneous cable subjected to its own weight (catenary) as proposed by Jwa et al. [73].

According to the INSST guide [15], electrical risk is defined as a circle around the cable, the radius of which is a function of the kV of the line. To define the risk zones along the BIM object with which the cable is modelled, concentric risk “cylinders” are generated in the BIM object, the axis of which is the cable itself. In this way, four risk zones are defined and subsequently modelled according to the criteria defined in the INSST guide [15] (danger distance 1 (DPEL-1, light red) and danger distance 2 (DPEL-2, dark red)). The presence of an unprotected worker poses a serious risk (DPEL-1 for lightning overvoltage and DPEL-2 without lightning overvoltage). Proximity distances 1 (DPROX-1, dark blue) and 2 (DPROX-2, light blue) limit the area around each danger zone, so that if no physical barrier is provided, the danger zones can be invaded. The diameters of the four are parameterized according to the OPL voltage given in [15].

To do this, a parameter is introduced to the element representing the OPL to introduce the nominal voltage of the line, which is of type number, and four parameters to define the diameter of the cylinders indicated above in fusion of the voltage. These diameters are obtained from a conditional formula relating the line voltage to the diameter [15]. This allows the OPL to be adapted to a multitude of projects, situations, and voltages. Finally, five new parameters are incorporated into the OPL BIM object to manage its visibility, so that the different elements that make up the OPL BIM object can be chosen to be seen or not in the model: four risk areas and the cable (Figure 1C).

### 3.3. Development of the Geometric Envelope of Machinery and Equipment

During the execution of a construction site, equipment and machines of different dimensions circulate under the OPL. Performing the entire risk analysis process in the design phase for each of their possible geometric configurations is a complex task, which can lead to errors. To simplify and automate the electrical risk analysis process, it is proposed to generate the analysis envelope for the machines considered in the project. Once the machines are known, they are introduced into the model as BIM objects, creating their contours with the main dimensions obtained from the manufacturer’s description (Figure 1D red).

Thus, for each BIM object of a machine, a BIM object is created which is the envelope of the geometry of that machine (Figure 1D green). To take into account that a machine can have different positions, the 3D envelopes of all the positions must be generated, which will be denominated as ($E^{e}\;M^{i}$) (*e* indicates the number of the position and *i* the number of the machine) (Figure 1D green and yellow). Thus, for example, the BIM object of position 1 of machine 1 will be named $E^{1}M^{1}$ (Figure 1D green). The BIM object of machine position 2 of machine 1 is $E^{2}M^{1}$ (Figure 1D yellow). In the creation of the envelope of each position, a safety guard is adopted according to the moving parts of the machine, such as cables, the position of which is difficult to determine. From the set of positions (*e*) of a given machine (*i*), the final envelope of that machine is obtained $M_{F}^{i}=\overset{j=e}{\underset{j=1}{\sum }}E^{j}\times M^{i}$, which contains the most unfavorable points of all the positions of machine *i* (Figure 1D blue).

Finally, from all final envelopes of each machine ($M_{F}^{i}$), the BIM object of the analysis envelope $M^{A}=\sum_{j=1}^{j=i}M_{F}^{j}$ resulting from all final envelopes of each machine *Mi* is created. This generalizes the risk assessment process for all machines and for the different work processes using the worst-case envelope (Figure 1D red).

In the construction phase, the same process must be carried out, updated with the information of the final machines to be used and their real dimensions.

### 3.4. 4D Collision Simulation and Analysis

In the Revit model with the geometry of the road, the BIM objects of the OPL supports, the parameterized cable with the risks and the machinery analysis envelope (*M^A^*) are introduced. In this model, the execution phases are created, varying both the positions of the machine and the layer of material to be executed according to the construction process of the road around the OPL.

For the electrical risk, the movement of the machines under the OPL is simulated by moving the BIM object *M^A^* along the road for each phase of embankment and pavement construction. In the simulation of the machine path, the horizontal and vertical position of the machine is evaluated. The horizontal position of the machine varies according to the section (distance between layers) it is running and its exact position within that section at each value. Given the geometry of the machines, sections less than 10 m from the line are analyzed, varying the horizontal position of the machine in this section in increments of 0.5 m. The vertical position of the machine varies according to the layer of material that is being executed. Four layers have been applied for the embankment and five for the road surface: two of granular material and three of bituminous mixes. The combination of the different horizontal and vertical positions simulates all the locations that the *M^A^* envelope can have with respect to the OPL.

In the process, many geometric checks must be performed. To facilitate these operations, as well as to avoid errors, the process has been automated using object-based programming with Dynamo. Being integrated into the Revit modelling platform, its use brings advantages over other object-based programming tools, as it extends the capabilities of Revit by being able to interact with it and manage its parameters also from external inputs from Excel [74]. Once developed, the Dynamo script is executed from Revit, which allows the risk manager to interact with the model and then query and change the properties of the elements in the same management tool [58].

The developed script allows the selection of the BIM objects of the road and the BIM object of the *M^A^* envelope, depending on parameters associated with the section, the material layer, execution phase, etc. For each execution phase, collision detection of the *M^A^* envelope with the four risk zones of the cable is performed (Figure 1E).

The collision analysis, in each section and for each material layer, is performed by exporting the Revit model to NavisWorks 2020, and using “Clash Detective” it is analyzed if the 3D geometry of the *M^A^* collides with the 3D geometry of the electrical risk zones of the OPL. The collision detection is automated using a Dynamo 2.3 script that generates a georeferenced sphere family representing the collision point in the Revit model (Figure 1F).

### 3.5. Electrical Risk Management in BIM

For each of the sections and layers of material on which the collision analysis has been performed, the risk assessment based on the INSST guide [15] is performed in accordance with BSI PAS1192-6:2018 [30]. The risk level is the combination of the severity of the damage and the probability of occurrence measured on a qualitative scale. To transform it into a quantitative scale, we follow what is proposed by Cortés-Pérez et al. [32]. Thus, the health and safety technician will apply for the probability (Prob) the value 1, 2, or 3 depending on whether it is low, medium, or high, respectively. The severity (Sev) can take the value 1, 2, or 3 depending on whether it is slightly harmful, harmful, or extremely harmful, respectively. The risk assessment (Val) is the product of probability and severity, resulting in a risk assessment matrix [32] with a color-coded risk level according to the INSST’s Occupational Risk Assessment method [75] (Table 1).

The electrical risk assessment of the OPL is carried out for each zone defined in Section 3.2. Given the type of work for which the risks are analyzed, vehicle traffic, it is considered that the collision of the *M^A^* with the OPL in the model gives rise to the risk of lightning overvoltage (DEP-1, dark red) and protection against electrical risk cannot be guaranteed. If the working area can be precisely delimited, it shall be considered as proximity zone 1 (DPROX-1, dark blue), and if not possible, it shall be proximity zone 2 (DPROX-2, light blue).

The quantification of the severity parameter shall take the value 1 if *M^A^* does not touch any of the risk zones, 2 if it invades one of the proximity zones, and 3 if it does so in the danger zone. The probability parameter will take the value 1 if does not touch any of the risk zones, 2 if it invades the proximity zone and 3 if it does so in the danger zone. The product of both parameters will give the electrical risk assessment which will be qualified according to (Table 1), as a result of which the necessary measures and protections will be applied as established [75].

For the introduction of preventive measures in the model, it is necessary to create BIM objects for signaling and marking in accordance with the regulations that regulate them, for example [72]. For the automatic application of these measures in the model, a Dynamo script was created. In it, from the coordinates of the collision point of *M^A^* with the risk cylinder, the location point of the sign or marking is obtained at the edge of the roadway and at the distance established by the standard, considering the specific geometry of the construction phase in which the analysis is carried out. At these points, the BIM objects of the signage and marking (Figure 1F) defined in accordance with the standard are inserted. If the risk is still intolerable even with signage and marking, the OPL must either be removed or the construction equipment or workflows must be changed, resulting in a new 4D of the project (Figure 1F).

## 4. Results

**Figure 2:**
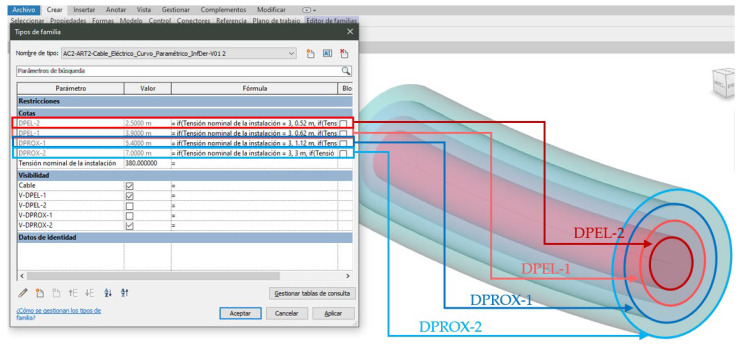
Creation of the parametric family of the electrical cable.

The application of the methodology, and therefore of the simulation, was carried out on the crossing of a 380 kV OPL on a straight section of a road junction project located in Miranda de Ebro (Spain) (Figure 1A). The investigation was based on the construction project with the complete layout, the topography, the geographical location of the OPL, its characteristics, and the works plan. This case study allows validation for the application of the research to other road projects, as it is a common case in road works and, furthermore, the general regulatory framework for Health and Safety at the European [4] and national [5] level was considered in its development, following its requirements for evaluation, preventive measures, and re-evaluation. The specific regulations on electrical risk assessment [14,15] were applied to determine the risk areas and their parameterizations (Figure 1C and Figure 2).

Finally, for the risk assessment within the BIM model, the methodology of the National Institute for Safety and Health at Work [75] is followed (Figure 1F) in accordance with the requirements of assessment, preventive measures, and reassessment.

Figure 1 shows the general workflow for the automation of OPL electrical risk detection, management, and analysis in road BIM projects. Starting from the road layout model and topography (Figure 1A), it is exported to Revit at the project coordinates, where the risk analysis is performed (Figure 1B).

With the information on the location and characteristics of the OPL that interferes with the project, in the Revit model of the road, the model of the OPL is generated, which in this case is composed of three cables (Figure 1C). Figure 2 shows the cables modelled from the balance equations [73], and the creation and parameterization of the risk zones of one of the cables according to the technical guide of the National Institute for Safety and Hygiene at Work [16], as well as the parameterization of their visualization for better information management. In the case of application, the OPL has a voltage of 380 volts, and the danger distances (DPEL-1 and DPEL-2) are obtained as cylinders with 3.90 and 2.50 m radius, respectively, and the cylinders with proximity distances 1 and 2 (DPROX-1 and DPROX-2) with a radius of 5.40 and 7.00 m, respectively.

Given the atmospheric characteristics of the area and the work in this case, it is considered that there is a risk of lightning overvoltage and the work area is not delimited, only the danger distance 1 (DPEL-1, light red) and the proximity distance 2 (DPROX-2, light blue) are considered (Figure 2, top right). To work with them, Figure 2 shows the visibility parameters leaving only DPEL-1 and DPROX-2 visible.

The next step in the electrical risk assessment is to obtain the final machine envelope from all the machinery in the project (Figure 1D). Table 2 shows the 3D geometry of the machines involved in this case study. The BIM objects (Mi) that simulate the machines used in the earthmoving and asphalt pavement spreading phase are: truck, loader, backhoe, backhoe loader, motor grader, paver, and road roller. The geometry envelope has been created for these BIM objects, considering a safety guard of 0.50 m to consider loose parts such as cables or hoses.

The case study involves six machines, of which three have two positions and the others have only one position. With two positions there is the loader that has been modelled with the bucket lowered (*E*^1^ *M*^1^) and raised (*E*^2^ *M*^1^), which gives the final envelope $M_{F}^{1}=\sum_{j=1}^{j=2}E^{2}\times M^{1}$. The same happens with the backhoe and the truck, obtaining the final envelopes $M_{F}^{4}$ y $M_{F}^{5}$. The six final envelopes are obtained in the same way and then the analysis envelope $M^{A}=\sum_{j=1}^{j=6}M_{F}^{6}$ is obtained (Table 2). This analysis envelope has a maximum length and height of 12.54 and 5.23 m, respectively.

**Figure 3:**
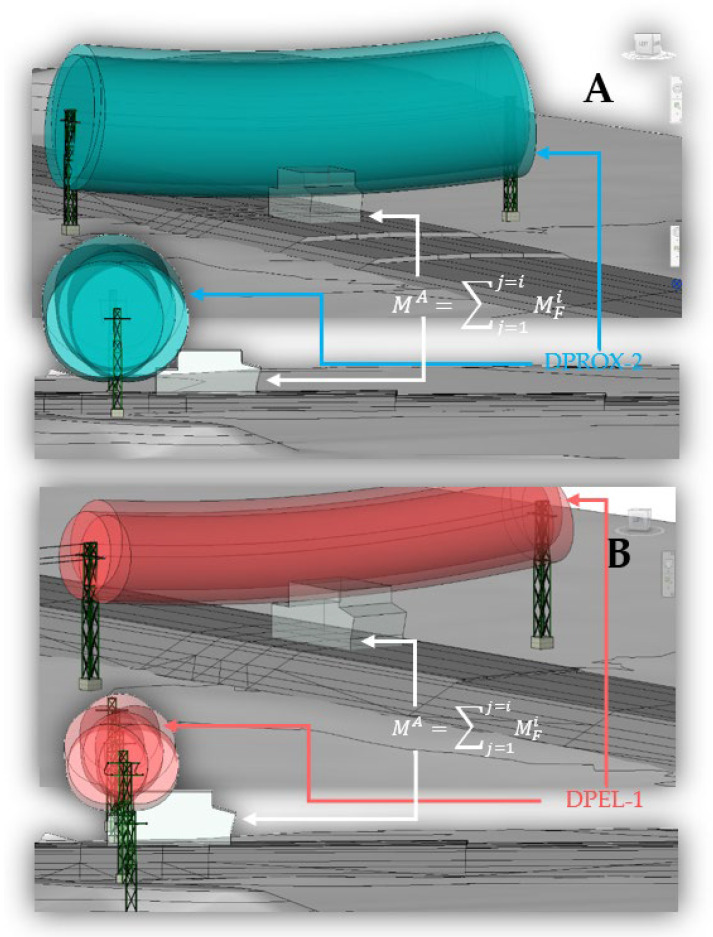
3D view and longitudinal view of the 4D simulation in Revit. (**A**) Paving phase with collision analysis with cable proximity zone 2. (**B**) Bituminous mix paving phase with collision analysis with cable danger zone 1.

The 3D model of the route, the topography, the cable with the risk zones, and the analysis envelope forms the model of the traffic risk analysis under the OPL. For the 4D simulation, the construction phases are created according to the project’s construction plan and the analysis envelope under the OPL is circulated in these construction phases (Figure 1E and Figure 3).

Figure 3 shows the simulation of the passage of the envelope in two different phases and with a different electrical risk zone. Part A (upper part of Figure 3) shows the 3D view and the elevation of the execution of the last layer of embankment material in the section just below the OPL. This simulates the variation of the horizontal position of the machine during the execution of this layer with proximity zone 2 (DPROX-2, light blue). Part B (lower part of Figure 3) shows the 3D view and elevation of the execution of the last HMA paving layer in the section just below the OPL, simulating the variation of the horizontal position of the machine during the execution with danger zone 1 (DPEL-1, light red).

**Figure 4:**
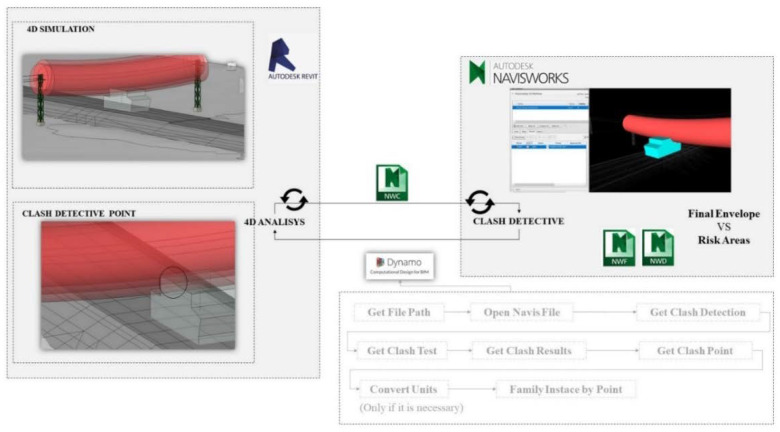
Collision analysis between the final envelope and hazard zone 2 in the HMA spreading phase, wearing course.

**Figure 5:**
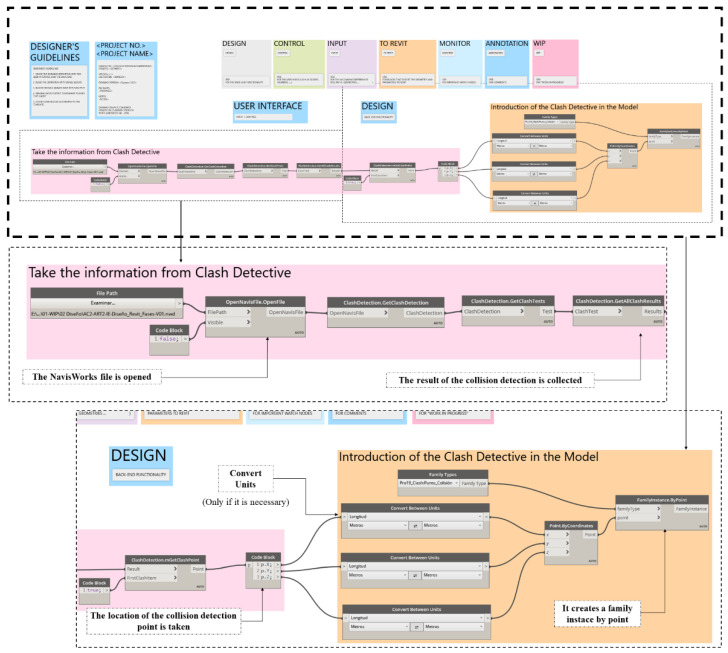
Image on top: general outline of the script. Intermediate image loading information from the point where the collision of the analysis envelope occurs. Bottom image: introduction of the collision point in the BIM model.

The 4D simulation model in the different phases of execution of the embankment and pavement in Revit is exported to NavisWorks in *.nwc format. In NavisWorks where the collision detection is performed, the position of the collision point is collected by means of a Dynamo script that introduces it into the Revit model for review and coordination (Figure 1E). The process is repeated cyclically for each of the analysis envelope positions and each of the execution phases. As an example, Figure 4 shows the analysis procedure for the collision detection analysis envelope with the risk areas in the HMA (Hot Mix Asphalt) paving phase. Starting with the export from Revit of the 4D simulation to NavisWorks where the collision analysis is performed using the “Clash Detective” tool (Figure 5). It shows the Dynamo script which is structured in two steps. The input requirements are the NavisWorks generated. nwd file that has the collision point information. The first part extracts the point. In the second part, the X, Y, and Z coordinates are extracted and, if necessary, a unit conversion is done to finally generate a point in Revit.

**Figure 6:**
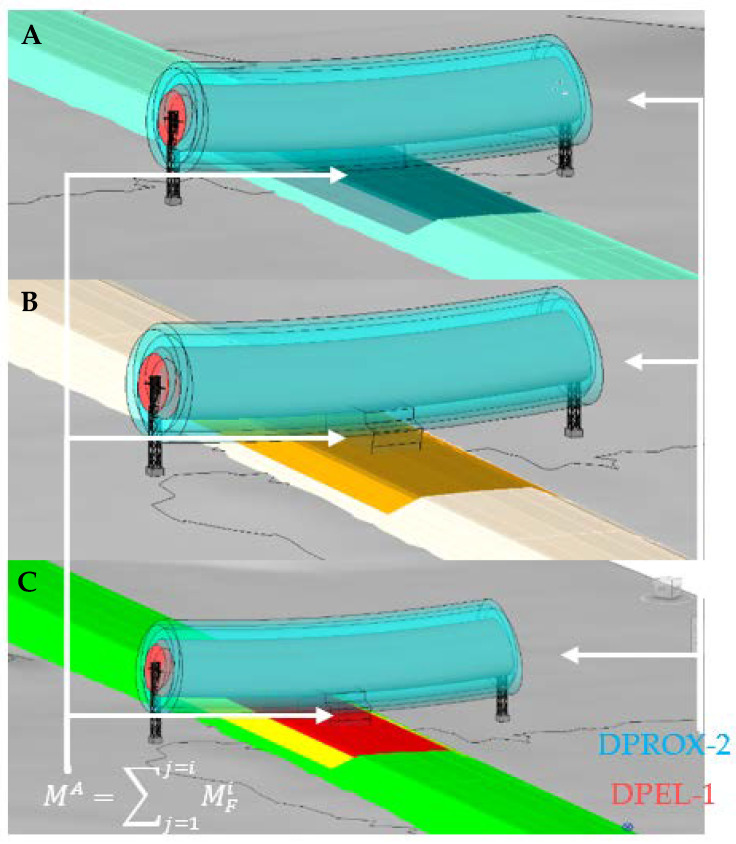
Risk assessment of the study area for the implementation of the embankment and road surface (color coded according to Table 1). (**A**) Risk probability (1 = light blue, 2 = medium blue, 3 = dark). (**B**) Risk severity (1 = light orange, 2 = medium orange, 3 = dark orange). (**C**) Risk assessment (1 = light green, 2 = dark green, 4 = yellow, 6 = orange, 9 = red). Note: Delimitation of the zones according to the diagram in Figure 2 and Figure 3.

The next step is the assessment of the level of risk on the construction site in the OPL environment (Figure 1F and Figure 6). To do this, it is necessary to analyze the probability, severity, and risk level of each of the situations generated. Figure 6 shows the result of the analysis of the embankment and pavement phases. The risk is trivial in all the sections and layers of material except for the sections closest to the OPL and the one just below it. It can also be seen that for these nearby layers, the level of risk increases from trivial (first three layers corresponding to the embankment), passing to a moderate risk (last layer of the embankment and two granular layers of the road surface) at the moment when the envelope begins to touch the proximity zone 2 (DPROX-2, light blue), to an intolerable risk (execution of the 3 HMA layers of the road surface) at the moment when the envelope begins to touch the danger zone 1 (DPEL-1, light red).

**Figure 7:**
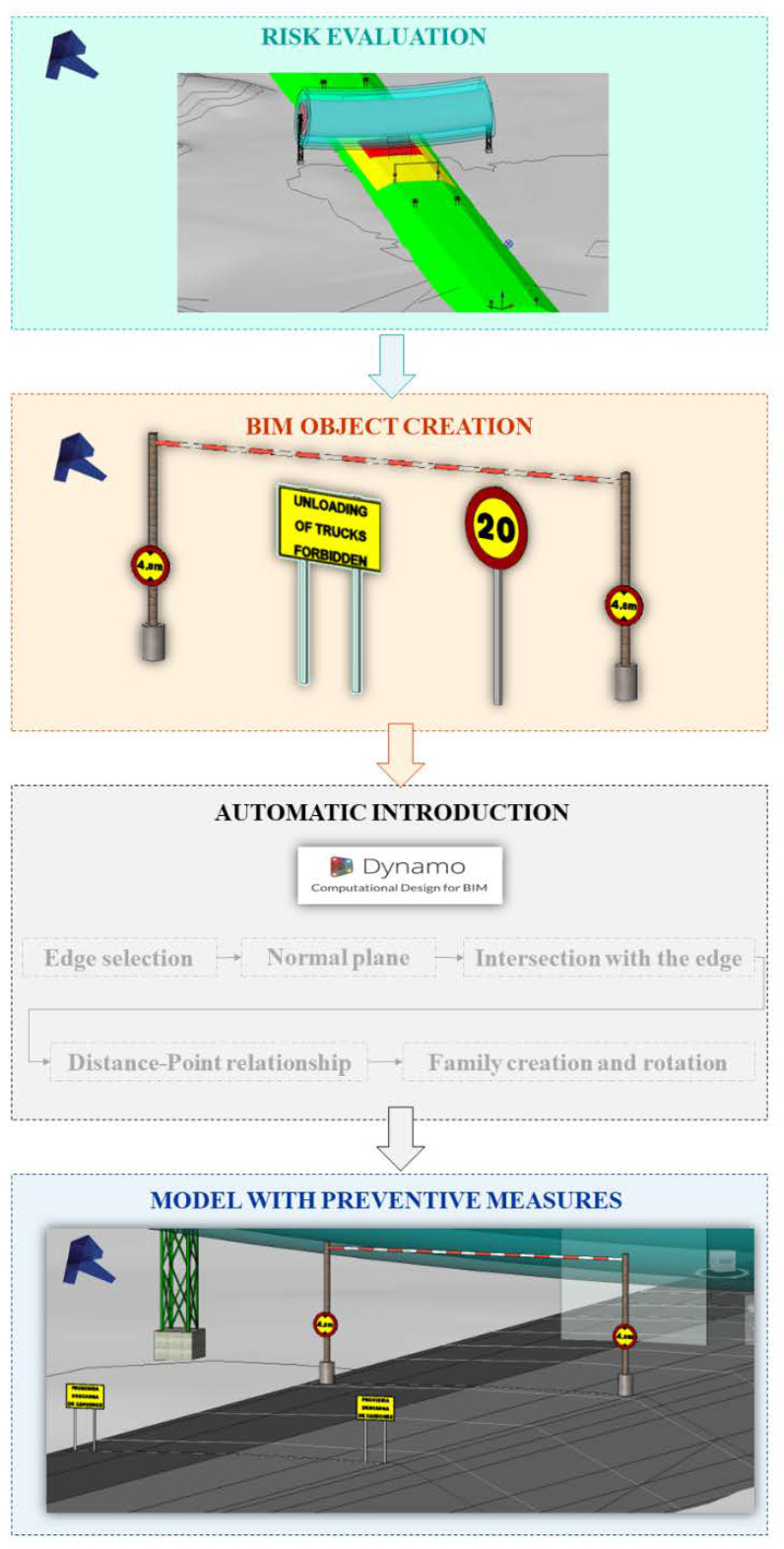
Creation of parametric BIM objects representing preventive measures according to the risk assessment and automation of their introduction into the model.

**Figure 8:**
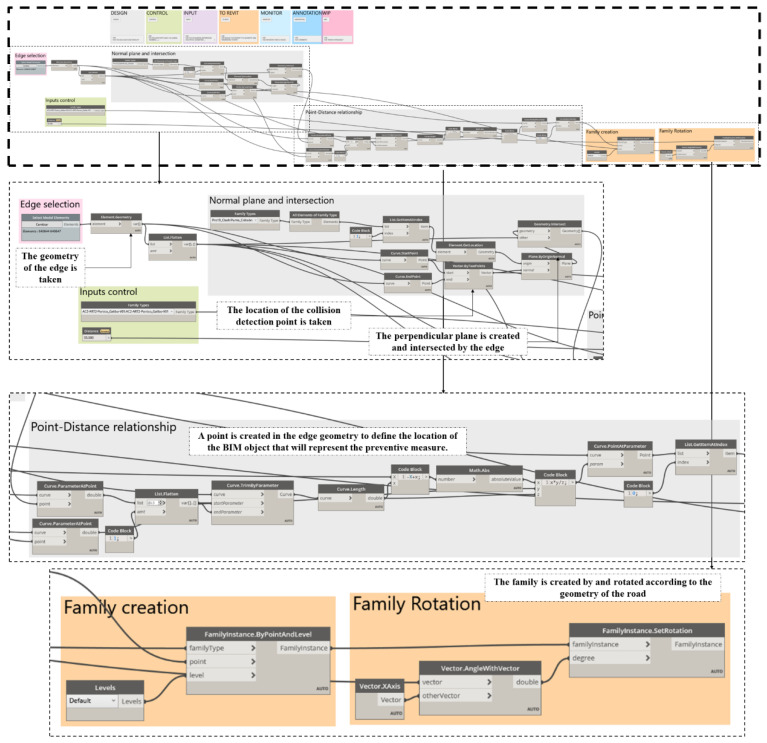
Script for automating the introduction of preventive measures in the model based on the risk assessment and the collision point between the analysis envelope of the machines and the risk zones.

Based on the risk assessment (Figure 6), the preventive measures to be applied in each phase of execution are determined in accordance with the standard [72] following the process proposed in Figure 7. The second image in Figure 7 shows the BIM objects created to represent the necessary preventive measures. The third image shows the general scheme of the Dynamo script, detailed in Figure 8, to insert the preventive measures automatically in the model, in their correct position according to the execution phase and the coordinates of the collision point obtained with the script of Figure 5. When carrying out the risk assessment in the case of the execution of the last layer of HMA of the road surface, given that an intolerable risk level is produced, it is necessary to incorporate two signs TR-301 limiting the speed to 40 km/h at 125 m from the intolerable risk zone; 2 signs TS-860 prohibiting the unloading of trucks at 25 m from the intolerable zone, and a gantry with a gauge limitation of 4.80 m, at vertical distance from the danger zone 1 (DPEL-1, light red) to the last layer of HMA is proposed. This is replicated on the other side in a symmetrical way.

Figure 8 shows the script for inserting the preventive measures in their correct position. It is structured in two code blocks, the first one takes the geometry of the collision point and the curve that represents the edge of the road in that phase of execution. Once obtained, a plane containing the collision point and perpendicular to the curve is created to obtain the intersection between the curve and the plane. In the second block, starting from this intersection point, the insertion point is created along this curve at the desired distance where the corresponding BIM object will be placed, rotating perpendicularly to it so that it is shown in front of the circulating vehicles. This process is applied for each type of sign, in this case it has been applied to introduce the vertical warning signs and the free height control gantry. The distances and signage shall be applied according to the signage requirements of the specific regulations.

**Figure 9:**
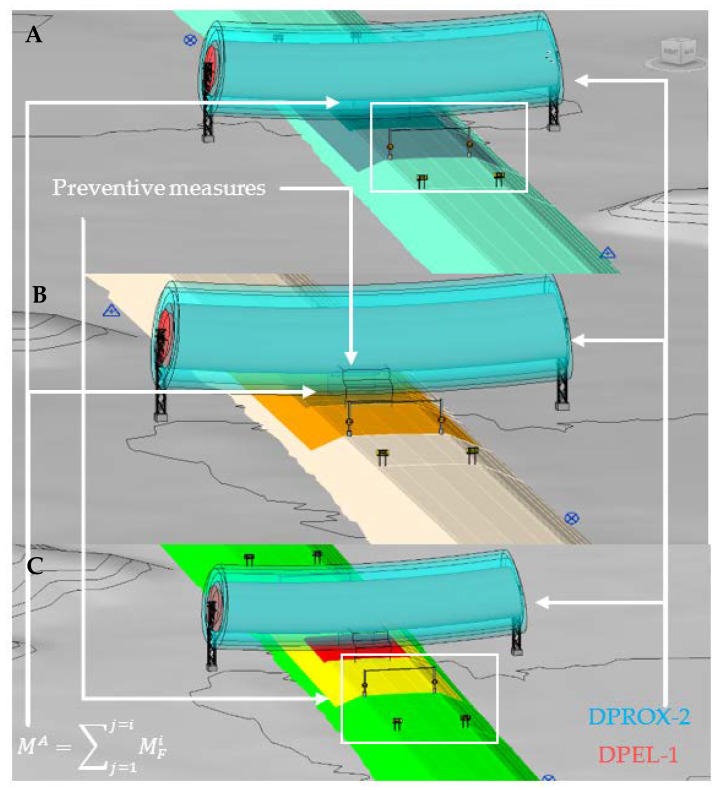
Risk reassessment of the study area (color coded according to Table 1). (**A**) Risk probability (1 = light blue, 2 = medium blue, 3 = dark). (**B**) Risk severity (1 = light orange, 2 = medium orange, 3 = dark orange). (**C**) Risk assessment (1 = light green, 2 = dark green, 4 = yellow, 6 = orange, 9 = red). Note: Delimitation of the zones according to the diagram in Figure 2 and Figure 3.

Once the measures are implemented, the risk is reassessed. Figure 9 shows the final risk severity and probability, determining the reassessed risk level. The trivial risk zones are maintained, but the arrangement of moderate and intolerable risk is changed by incorporating the signage prohibiting the unloading of trucks (limiting the position of the envelope) and the limitation of the height of the machines into the model. The risk remains moderate even in the execution of the HMA layers in the sections close to the OPL, thus reducing the risk.

**Figure 10:**
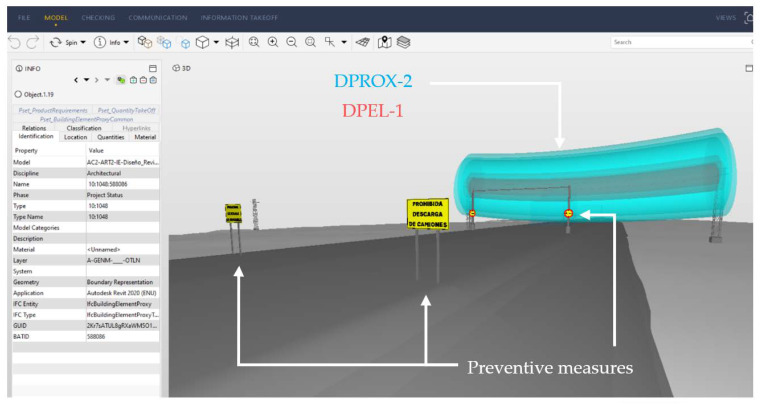
Exporting of preventive measures to IFC for coordination into the project.

Finally, Figure 10 shows the export to IFC of the preventive measures for coordination within the project leaving a traceable record of the decision making.

## 5. Discussion

The danger linked to the construction sector [1] and, in particular, the electrical risk [9] or [10] makes the concept of PtD become more and more important every day, based on avoiding or mitigating the hazards that lead to accidents through decisions taken in the design phase, based on a risk assessment of each component of the deliverable [31]. The proposal developed in this research based on BIM technology is in this line, allowing to perform a 3D analysis between machinery and OPL, in all phases of execution, providing a significant advance over current methodologies, improving the simulation, analysis, and assessment of electrical risk in the design phase, a risk that has been shown to be important in construction [8], treating it at the component level (Figure 1).

Authors such as Jin et al., indicate the importance of BIM methodology for health and safety management [61]. This study evidences this issue by presenting a proposal on how to improve electrical risk management based on BIM as a working methodology. Likewise, the automation of part of the process was introduced with the consequent reduction of analysis times and the minimization of human errors, a critical point to reduce risks in the design phase [59].

The proposed methodology represents a significant advance in the application of the regulations [14] and the technical guides [15] on electrical risk assessment, as they describe the risks using 2D models, which are difficult to apply and reflect the complexity of the three-dimensional phenomenon of the interaction of the geometry of machinery with electrical risk zones. By integrating the requirements of these standards into a 3D model in this study, it is possible to address any type of machinery-risk zone interactions, even the most complex ones, such as machinery or equipment that does not run perpendicular to the line path or running on non-horizontal platforms.

Just as Zhang et al. [63] integrated the regulatory requirements for fall-through openings or [64] safety assessment using verification rules, both in buildings, in this research the risks have been integrated into a linear model such as a road. This has been done by parameterizing the risk zones of the BIM object (Figure 2), as well as in the detection of the collision of the line with the design envelope.

By implementing the criteria of the health and safety regulations [5] and integrating the assessment methodology into the model itself [75], with the color code shown in Table 1, it is possible to comply with the legal requirements for risk assessment and its integration of the BIM methodology [29], enabling full integration in road projects carried out with BIM. This will allow obtaining the general advantages pointed out by Aires et al. [59], in addition to the specific ones indicated by [60] when carrying out simulations and by Azhar et al. [2] when using 3D models for the case of road projects, whose benefits have been demonstrated by Cortés Pérez et al. [32] in the case of buildings, representing a fundamental step towards establishing the data structure and specific processes required for BIM in linear infrastructures as indicated by Dikbas et al. [43], facilitating the integration of health and safety in strategies such as those of Great Britain [25] or Germany [26].

The parameterization of the risks carried out and the analysis of machine-design envelope interaction contemplates the risk controllers proposed by Malekitabar et al. [35] for the management in BIM of risks due to power lines, considering different positions of the machinery, as was carried out. However, this research represents a significant advance over that of Malekitabar et al. [35] by proposing to define an analysis envelope that collects the different positions of the machines. This facilitates analysis, but also management, by being able to define analysis envelopes according to the machinery in each phase of execution, making it possible to redesign site planning according to a detailed risk analysis, as proposed by Vahdatikhaki et al. [69], only for the earthmoving phase, extending the analysis in this study to the phases of pavement construction.

The proposal incorporates how to develop 4D simulation for risk analysis in different phases of execution, taking advantage of the benefits of BIM for the planning of construction tasks [63]. Thus, in each phase the complete risk assessment is carried out according to the INSST guide [75] in compliance with regulations [5]. This intimately connects safety with the work plan, allowing to know in each phase the level of risk and the measures to be applied, even allowing to decide, from the point of view of health and safety, which is the phase in which it is more appropriate to remove the line, as proposed by Raza et al. [49]. In fact, in the event that once the risk has been assessed, the preventive measures have been implemented in the model and the risks have been reassessed, the risk remains intolerable, a possible solution would be to carry out a new 4D of the project (Figure 1F). This validates the research done by Costin et al. [48] by allowing an optimization of the 4D planning, in addition to its visual simulation. This simulation allows different angles of the machinery to be considered, as proposed by Lou et al. [47].

To overcome the difficulty and uncertainty at the design level of risk analysis for the different geometries of each accident-causing phase and each machine that may circulate, the Zhang methodology [63] for the risk of falling through gaps has been used in the detection of risks. For this purpose, it has been proposed to create a BIM object that is the envelope of the construction machinery (Table 2), considering its different positions. Figure 3 shows the simulation in the different stages of the execution of the works following the construction logic and Figure 4 shows the collision analysis between the machinery envelope (Table 2) and the cable areas (Figure 2) following logical rules (Figure 4) as proposed by [50], but for the case of buildings and elements with less spatial complexity. In the execution phase, the final envelope can be obtained for each execution phase, for example, differentiating between the machinery in the earthmoving phase and that in the pavement execution phase, which allows a more precise analysis of the risks associated with the 4D of the actual execution of the work. An improvement on that proposed by Bae et al. [50] is the envelope concept that facilitates the management of geometries and risks of the same type and in the same area.

The proposed risk assessment integration methodology is an advance over that presented by Shen et al. [65] and Fernández et al. [66] as, although it describes the linking of a risk database to a BIM model with a color code, it does not perform the process of risk analysis, reassessment, and management within the model, as shown in Figure 6, Figure 7 and Figure 9. It is also a significant advance over Cortés-Pérez et al. [32] who, although they do carry out such assessment and reassessment work, it is carried out on building construction projects and not on linear infrastructure such as roads.

Other authors such as Getuli et al. [64] focus their study on the creation and standardization of health and safety BIM objects such as fences, which in this research have been developed to validate compliance with health and safety regulations [4,5], or [13] and perform risk reassessment (Figure 9), thus leaving the traceability of the project and being able to export all the information to an open format (IFC) for coordination within the project (Figure 10).

Through the parametric design employed, it is possible to adapt the methodology to different voltage ranges of the line, and to implement more refined analyses of the risk zones as proposed by Zhang et al. [33].

The proposed methodology, and the structure of the associated database, allows a step towards the integration of electrical risk assessment within Construction 4.0 through BIM, noting the advantages that [20,21] proposed for BIM and Construction 4.0. For example, in terms of geometry, although the starting point is a theoretical geometry of the cable, it is possible to define the real geometry by digitizing the line, as developed by Wang et al. [56] and Guo et al. [54], defining the real geometry with greater precision, thus being able to generate centimeter-accurate models [50]. The BIM model obtained through this methodology would be complemented with that proposed by Vahdatikhaki and Hammad [69], so that the information related to the position of these machines and their movements would be provided by the sensors installed on them. Thus, the risks of the machinery would be analyzed with real information, within the 3D model itself, taking advantage of the digitized terrain, the road, and the OPL, updating it and providing an input to, if necessary, act on the work planning, giving rise to a new 4D of the project.

The parameterization established at the OPL BIM object level and in the creation of the BIM model allows its adaptation for risk analysis in the construction phase. In this way, a highly detailed model can be generated by integrating it with the terrain models obtained with LiDAR [51], which can be applied in site monitoring using UVS [52], by integrating the real definition of the OPL with LiDAR and its transformation into objects [54].

Therefore, the implementation of this proposal will lead to a reduction in OPL electrocution accidents and associated delays, as the analysis integrates information, the absence of which causes accidents, such as vehicle geometry [35], non-visible arc flash hazard zones [33], and the accuracy of OPL routing [38] and its definition in drawings [40], especially vertical information [41].

Based on the parameterization developed, it is possible to use the proposed methodology for electrical risk management in line maintenance tasks or activities that take place in their environment, such as roads, agricultural or forestry work, and the machinery associated with them, representing an advance in the risk analysis of OPL [56] by integrating the vehicles that circulate in the lines.

Another line of work for the future is to extend this research to the construction of roads where, in addition to the activities and machinery discussed here, cranes are involved due to their relevance in terms of electrical risk and the complexity of their movements, extending the research to agricultural activities as well.

## 6. Conclusions

This research shows how to generate the BIM model of electrical risk analysis of an OPL that interferes with the construction of a road, from the digital model of the terrain and the road, incorporating the parametric model of the OPL and the model of the cables and the associated risk areas. From which all the health and safety information is integrated into a single model coordinated with the construction model.

Thanks to the parameterization of the BIM objects and the proposed methodology, the geometry of the OPL and its risk zones can be precisely defined, delimiting the work zones according to the type of machinery. In addition, the introduction of the machine analysis envelope concept allows the development of a detailed and accurate electrical risk analysis for all types of machinery or those specific to a construction phase, which facilitates 4D simulation and thus accurate electrical risk management during construction. This will aid decision making about the machines to be used and site planning, as well as determine when to eliminate the risk (remove the OPL), or if the risk cannot be eliminated, what signage and safeguards to put in place.

In addition, the automation of OPL risk detection, analysis, and management in BIM road projects allows eliminating or minimizing errors in risk analysis, establishing consistency between the different phases of risk assessment and traceability of decisions.

By generating a virtual model with structured information that can be exported in open format (IFC), the methodology makes it easier for all actors involved to interact in the collaborative environment for decision making related to the project’s risk management with respect to OPL.

All of this makes it possible to comply with the mandatory health and safety regulations for road projects developed with BIM with OPL interference. This will facilitate the analysis of electrical risk in the design phase, which will provide both the benefits indicated by the PtD, and the application of BIM in the analysis and management of occupational health and safety, to reduce the accident rate on construction sites.

In addition, the proposal can also be applied in risk management around the OPL during maintenance tasks or in agricultural works. 

## Figures and Tables

**Table 1:** Quantitative matrix of risk assessment in the BIM. The evaluated risk rating is in brackets.

		SEVERITY (Sev_R0i)		
		1 (Slightly Harmful)	2 (Harmful)	3 (Extremely Harmful)
PROBABILITY (Prob_R0i)	**1 (Low)**	1 (trivial risk-T)	2 (tolerable risk TO)	3 (moderate risk-MO)
	**2 (Medium)**	2 (tolerable risk-TO)	3 (moderate risk-MO)	6 (significant risk-I)
	**3 (High)**	3 (moderate risk-MO)	6 (significant risk-I)	9 (intolerable risk-IN)

**Table 2:** Creation of the machinery envelope. Around the image of the machine the perimeter of the envelope of each position is drawn.

	Number of Machines					
**Geometry in 3D**	$M^{1}$ 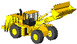	*M* ^2^ 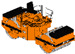	*M* ^3^ 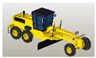	*M* ^4^ 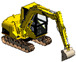	*M* ^5^ 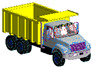	*M* ^6^ 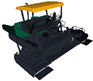
**Nº** **position**						
**1**	$E^{1}M^{1}$ 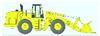	$E^{1}M^{2}$ 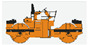	$E^{1}M^{3}$ 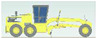	$E^{1}M^{4}$ 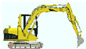	$E^{1}M^{5}$ 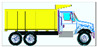	$E^{1}M^{6}$ 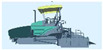
**2**	$E^{2}M^{1}$ ^ 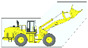 ^	-	-	$E^{2}M^{3}$ 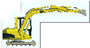	$E^{2}M^{5}$ 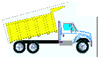	-
***e***	^…^	^…^	^…^	^…^	^…^	^…^
**Final** **envelope**	$M_{F}^{1}=\sum_{j=1}^{j=e}E^{j}\times M^{1}$	$M_{F}^{2}=\sum_{j=1}^{j=e}E^{j}\times M^{2}$	$M_{F}^{3}=\sum_{j=1}^{j=e}E^{j}\times M^{2}$	$M_{F}^{4}=\sum_{j=1}^{j=e}E^{j}\times M^{i}$	$M_{F}^{5}=\sum_{j=1}^{j=e}E^{j}\times M^{i}$	$M_{F}^{6}=\sum_{j=1}^{j=e}E^{j}\times M^{i}$
$M^{A}=\sum_{j=1}^{j=i}M_{F}^{i}$ 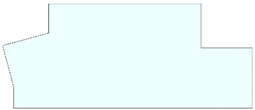			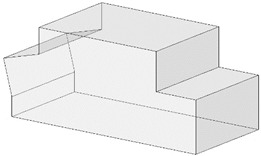			

## Data Availability

Not applicable.
